# The Regulation and Role of c-FLIP in Human Th Cell Differentiation

**DOI:** 10.1371/journal.pone.0102022

**Published:** 2014-07-14

**Authors:** Minna K. Kyläniemi, Riina Kaukonen, Johanna Myllyviita, Omid Rasool, Riitta Lahesmaa

**Affiliations:** 1 Turku Centre for Biotechnology, University of Turku and Åbo Akademi University, Turku, Finland; 2 National Doctoral Programme in Informational and Structural Biology, Åbo Akademi University, Turku, Finland; University of Oslo, Norway

## Abstract

The early differentiation of T helper (Th) cells is a tightly controlled and finely balanced process, which involves several factors including cytokines, transcription factors and co-stimulatory molecules. Recent studies have shown that in addition to the regulation of apoptosis, caspase activity is also needed for Th cell proliferation and activation and it might play a role in Th cell differentiation. The isoforms of the cellular FLICE inhibitory protein (c-FLIP) are regulators of CASPASE-8 activity and the short isoform, c-FLIP_S_, has been shown to be up-regulated by IL-4, the Th2 driving cytokine. In this work, we have studied the expression and functional role of three c-FLIP isoforms during the early Th cell differentiation. Only two of the isoforms, c-FLIP_S_ and c-FLIP_L_, were detected at the protein level although c-FLIP_R_ was expressed at the mRNA level. The knockdown of c-FLIP_L_ led to enhanced Th1 differentiation and elevated IL-4 production by Th2 cells, whereas the knockdown of c-FLIP_S_ diminished GATA3 expression and IL-4 production by Th2 cells. In summary, our results provide new insight into the role of c-FLIP proteins in the early differentiation of human Th cells.

## Introduction

T helper (Th) cells have an important role in body's defense against extra- and intracellular pathogens. Naive Th precursor (Thp) cells become activated by T cell receptor (TCR) signals from an antigen presenting cells and their polarization to different Th subtypes is dependent on the cytokine milieu as well as co-stimulatory factors presented by the antigen presenting cells. Different Th subtypes are characterized by the expression of different transcription factors, cell surface receptors and the secretion of cytokines. The first-characterized and most widely studied subtypes are Th1 and Th2 cells, which are important for cell-mediated immunity eradicating intracellular pathogens and humoral responses, respectively. If uncontrolled, Th cells can mediate immunopathology, such as asthma and autoimmune diseases like Type 1 Diabetes.

TCR activation leads to the activation of several pathways, such as Ras/extracellular signal-regulated kinase (ERK), Nuclear factor of activated T cells (NFAT) and Nuclear factor kappa enhancer binding protein (NF-kB) pathways, which are important for the initial activation and for the ability of T cells to differentiate into functional subtypes. However, in addition to TCR activation, cytokines interleukin-12 (IL-12) and IL-4 are crucial for driving the differentiation of Th1 and Th2 cells, respectively. IL-12 and interferon-γ (IFNγ) as well as transcription factors STAT4, STAT1 and T-Box expressed in T cells (TBET) are the main factors involved in Th1 cell differentiation [1]. Naive Thp cells secrete IFNγ in response to TCR activation, which is mediated by NFAT and NF-κB transcription factors [2], [3]. IFNγ induces the differentiation of Th1 cells through STAT1 signaling [4]. These signaling pathways then lead to the expression of TBET [5], [6]. TBET is required for IL-12 receptor β2 (IL-12Rβ2) expression, thus making the cells responsive to IL-12 [7]. IL-12Rβ2 expression is maintained by IFNγ signaling [7], [8]. Once the expression of IL-12Rβ2 is up-regulated, IL-12 is able to activate STAT4, an important inducer of IFNγ and IL-12Rβ2 expression [9]–[12].

IL-4 signaling through IL-4 receptor (IL-4R) activates signal transducer and activator of transcription (STAT) 6, which is a key transcription factor for Th2 responses [13]. The importance of IL-4 and STAT6 for Th2 differentiation has been shown with *Il-4^-/-^* and *Stat6^-/-^* mice, which have impaired Th2 differentiation [14], [15]. STAT6 and IL-4 induce the expression of GATA binding protein 3 (GATA3) transcription factor, which is important for appropriate Th2 differentiation and IL-4 secretion by Th2 cells [1], [16]. GATA3 is also able to activate its own expression in a STAT6-independent manner [17]. Th1 and Th2 transcription factors, TBET and GATA3, are also able to suppress the differentiation of the other subtypes both by indirect and direct manner [6], [18]–[20].

In addition to cytokines and co-stimulatory molecules, T cell development is also regulated by caspase pathways, which usually regulate programmed cell death, i.e. apoptosis [21]. Cellular FLICE inhibitory protein (c-FLIP, gene name *CFLAR*) is a regulator of CASPASE-8 activity and has also been shown to regulate NF-κB and ERK signaling pathways [22], [23]. c-FLIP has several isoforms detected at the mRNA level, but only three of them are expressed at the protein level [24], [25]. All of the c-FLIP isoforms, c-FLIP short (c-FLIP_S_), c-FLIP raji (c-FLIP_R_) and c-FLIP long (c-FLIP_L_) function as anti-apoptotic molecules and inhibit caspase-8 activity [25]–[27]. c-FLIP_L_ is a homologue of caspase-8 and has an inactive caspase domain in the C-terminal end [28], whereas the two short isoforms lack the caspase-like domain [26]. The inhibition of caspase-8 activity leads to the increased production of IL-4 and enhanced Th2 polarization [29] and in line with this, our previous results indicated IL-4 as a possible regulator of c-FLIP_S_ expression [30]. In addition, c-FLIP_L_ transgenic mice have a more profound Th2 phenotype [31]. TCR activation has previously been shown to up-regulate especially c-FLIP_S_ thus protecting the cells from apoptosis [32], [33]. c-FLIP expression is also regulated by several transcription factors including NF-κB and NFAT as well as ERK/Mitogen activated protein kinase and Phosphatidyl-inositol 3 kinase/Akt signaling pathways [34]–[37]. In addition, c-FLIP has been shown to inhibit T-cell activation [38] as well as to regulate NF-κB and ERK signaling [22]. However, the role and expression of c-FLIP isoforms have not previously been studied during human Th1 or Th2 cell differentiation.

In this paper, we have studied the expression of c-FLIP isoforms during the differentiation of human Th cells and their role in this process. RNA interference was exploited to study the role of c-FLIP_S_ and c-FLIP_L_ in Th1 and Th2 differentiation. Our results indicate that down-regulation of c-FLIP_L_ increased the proliferation but also the number of apoptotic cells during the early differentiation of human Th1 and Th2 cells. In addition, c-FLIP_L_ knockdown enhanced the expression of Th1 marker genes but also the production of IL-4 by Th2 cells, whereas the depletion of c-FLIP_S_ down-regulated both IL-4 production and GATA3 expression by Th2 cells. This study provides new insight into the roles of c-FLIP proteins in human Th cell differentiation.

## Materials and Methods

### Cell culture and transfections

Human mononuclear cells were isolated from the cord blood of healthy neonates using Ficoll-Paque isolation (Amersham Pharmacia Biotech, Uppsala, Sweden). Positive isolation with DYNAL magnetic beads (Invitrogen, Carlsbad, CA) was used to further purify CD4+ cells. Cells from several individuals were pooled after the isolation. Yssel's medium (IMDM [Invitrogen] supplemented with Yssel medium concentrate [39], pen/strep and 1% AB-serum) was used for culturing of the cells. Plate-bound α-CD3 (0.125 µg/well) and soluble α-CD28 (0.5 µg/ml; both from Immunotech, Marseille, France) were used for activation and at the same time cells were polarized towards Th1 direction with 2.5 ng/ml of IL-12 or Th2 direction with 10 ng/ml of IL-4 (both from R&D Systems, Minneapolis, MN) or cultured without addition of cytokines (Th0 cells). IL-2 (40 U/ml, R&D Systems) was added into all of the cultures after 48 h of priming.

For c-FLIP_S_ and c-FLIP_L_ knockdown experiments, freshly isolated CD4+ cells were suspended in Optimem I (Invitrogen) and transfected with small interfering RNA (siRNA) oligonucleotides (Sigma-Aldrich, St Louis, MO) (Table 1) using the nucleofection technique (Lonza, Basel, Switzerland). 4×10^6^ cells were transfected with 1.5 µg of siRNA (non-targeting (NT), c-FLIP_S_, c-FLIP_L_ or STAT6 targeting siRNA). The transfected cells were allowed to rest for 20–24 h in RPMI 1640 medium (Sigma-Aldrich) supplemented with pen/strep, 2 mM L-glutamine and 10% FCS at 37°C (2×10^6^ cells/ml) and subsequently activated and cultured in Yssel's medium as described earlier in this section.

**Table 1:** Sequences of primers, probes and siRNA oligonucleotides used.

Taqman RT-PCR	
c-FLIP_L-Probe	Universal probelibrary #14 (Roche)
c-FLIP_L-F	5'-GCTCACCATCCCTGTACCTG-3'
c-FLIP_L-R	5'-CAGGAGTGGGCGTTTTCTT-3'
c-FLIP_R-Probe	5'-6(FAM)-CCAGACTCACCCTGAAGTTATTTGAAGGATCCT-(TAMRA)-3'
c-FLIP_R-F	5'-CAAGCAGCAATCCAAAAGAGTCT-3'
c-FLIP_R-R	5'-TCATGCTGGGATTCCATATGTTT-3'
c-FLIP_s-Probe	5'-6(FAM)-TTCAGGATGATAACACCCTATGCCCATTGTC-(TAMRA)-3'
c-FLIP_s-F	5'-TCTCCAAGCAGCAATCCAA-3'
c-FLIP_s-R	5'-TCACATGGAACAATTTCCAAGAATTTT-3'
EF1a-Probe	5′ -6(FAM)-AGCGCCGGCTATGCCCCTG-(TAMRA)- 3′
EF1a-F	5′ -CTGAACCATCCAGGCCAAAT- 3′
EF1a-R	5′ GCCGTGTGGCAATCCAAT- 3′
Gata3-Probe	5'-6(FAM)-TGCCGGAGGAGGTGGATGTGCT-(TAMRA)-3'
Gata3-F	5'-GGACGCGGCGCAGTAC-3'
Gata3-R	5'-TGCCTTGACCGTCGATGTTA-3'
IFNg-Probe	5' -6(FAM)-TGCTGGCGACAGTTCAGCCATCAC -(TAMRA)- 3'
IFNg-F	5' -TGTCCAACGCAAAGCAATACA- 3'
IFNg-R	5' -CTCGAAACAGCATCTGACTCCTT- 3'
IL-12Rb2-Probe	5' -6(FAM)-TGCATTGCTATCATCATGGTGGGCAT-(TAMRA)- 3'
IL-12Rb2-F	5' -CGTTTGTGGCACCAAGCA- 3'
IL-12Rb2-R	5′ -GCTGGAAGTAATGCGTTGAGAA- 3′
T-bet-probe	5′ -6(FAM)-TCAGCATGAAGCCTGCATTCTTGCC-(TAMRA)- 3′
T-bet-F	5′ -ACAGCTATGAGGCTGAGTTTCGA- 3′
T-bet-R	5′ -GGCCTCGGTAGTAGGACATGGT- 3′

siRNA sequence	
Non targeting (NT)	5′ -GCGCGCUUUGUAGGAUUCG- 3′
c-FLIP_Long siRNA	5′-GAGCUUCUUCGAGACACCUUC-3′
c-FLIP_Short siRNA	5′-CACCCUAUGCCCAUUGUCCU-3′
STAT6	5′ -AAGCAGGAAGAACUCAAGUUU- 3′

F indicates forward; R indicates reverse; FAM, *6-carboxyfluorescein*; and TAMRA, *6-carboxyltetramethylrhodamine*.

### Ethics statement

Research involving the use of blood from anonymous donors was permitted by the Ethics Committee of the Hospital District of Southwest Finland (permission granted 24.11.1998; article #323). An oral informed consent was obtained from the mothers of neonates and the use of oral consent was approved by the Ethics Committee. The blood was collected from the umbilical cord after delivery and the samples were collected and handled anonymously.

### Real-time quantitative RT-PCR

Total RNA was isolated and samples were prepared for RT-PCR analysis as previously described [40]. Gene expression levels were measured using the TaqMan ABI Prism 7900HT Sequence Detection System (Applied Biosystems, Foster City, CA) [41]. The primers and probes used (Oligomer, Helsinki, Finland) (Table 1) were designed using Primer Express software (Applied Biosystems) or ProbeFinder software (Roche, Mannheim, Germany) for Universal Probe Library assays (Roche). The mRNA levels were normalized against the levels of a housekeeping gene elongation factor 1 alpha (EF1α) [41].

### Western blotting

Cells were lysed in Triton-X-100 lysis buffer (TXLB; 50 mM Tris-HCl pH 7.5, 150 mM NaCl, 0.5% Triton-X-100, 5% glycerol, 1% SDS, 1 mM Na_3_VO_4_, 10 mM NaF) or SDS lysis buffer (62.5 mM Tris-Hcl (pH 6.8), 2% (w/v) SDS, 10% glycerol, 50 mM DTT, 0.1% (w/v) bromphenol blue), boiled for 5 minutes and sonicated. Subsequently equal amounts of protein were separated by SDS-PAGE electrophoresis and transferred to nitrocellulose or PVDF membranes. The proteins studied were detected using the following primary antibodies: mouse α-cFLIP (NF6: Alexis Biochemicals, Lausanne, Switzerland), mouse α-STAT6 (BD Biosciences, San Jose, CA), mouse α-GAPDH (#5G4, 6C5, HyTest, Turku, Finland) or mouse α-β-ACTIN (Sigma-Aldrich). Horseradish peroxidase-conjugated goat α-mouse IgG (SC-2005; Santa Cruz Biotechnology, Santa Cruz, CA), goat α-mouse IgG1 (Southern Biotech, Birmingham, AL) or α-rabbit IgG (BD Biosciences) were used as secondary antibodies. The proteins were visualized with enhanced chemiluminesence (GE Healthcare), and quantified with a microcomputer imaging device (MCDI; M5+, Imaging Research Inc., St. Catharines, Canada) or with ImageJ [42] and normalized against β-ACTIN or GAPDH.

### Measurement of cell proliferation

To study the proliferation of transfected Th1- or Th2-polarized cells, cells were transfected as described earlier in cell culture and transfections section. Cells were harvested 20 h after transfection, washed twice with PBS and re-suspended in 2.5 µM carboxyfluorescein succinimidyl ester (CFSE; Invitrogen) in 5% FCS/PBS (w/v) and incubated for 10 min at RT. The labeling was stopped with 10× volume of 5% FCS/PBS (w/v) and cells were washed twice with 5% FCS/PBS. CFSE labeled cells were then cultured under Th1 or Th2 conditions for 48 to 96 h as described in the Cell culture and transfections-section. The CFSE staining of the cells was measured by FACSCalibur system and analyzed with CellQuest Pro (both from BD Biosciences) or FlowJo (TreeStar Inc., Ashland, OR, USA). The proliferative index was calculated as the sum of the cells in all generations including the parental divided by the calculated number of original parent cells theoretically present for each time-point and sample.

### Flow cytometry

To study the apoptosis of transfected Th1 and Th2 polarized cells, cells were transfected, rested for 20–24 h and cultured for 24 h or 48 h as described earlier in the Cell culture and transfections-section. 0.5×10^6^ cells per sample were then harvested, washed twice with PBS and once with 1xBinding Buffer ((5 mM HEPES, 70 mM NaCl, 2.5 mM CaCl_2_, pH 7.4) in 2%FCS/PBS (w/v), 0.01% NaN_3_). Cells were stained with Annexin V-FITC (BD Pharmingen, San Jose, CA) and incubated at RT for 20 min. Cells were then washed twice with Binding buffer. 20 s prior to analysis with FACSCalibur system, propidium iodide (PI; BD Pharmingen) was added to the sample. The data was analyzed with CellQuest Pro (BD Biosciences) or FlowJo (TreeStar Inc).

For CD69 analysis, transfected cells were cultured for 24 h in Th1 or Th2 polarizing conditions and 0.5×10^6^ cells per sample were harvested for staining. Cells were washed with 2% FCS/PBS, 0.01% NaN_3_ and stained with CD69-FITC (BD Biosciences) or isotype control anti-mouse IgG1-FITC (MG101, Invitrogen). Cells were analyzed with the FACSCalibur system and analyzed with CellQuest Pro (both from BD Biosciences).

### Bio-plex Cytokine assay

To measure IFNγ produced by Th1 polarized cells, duplicate samples were stained on 96-well plates according to the manufacturer's instructions (Milliplex Map Kit (assay sensitivity: minimum detectable IFNγ concentration  = 0.8 pg/ml); Millipore, Billerica, MA) and measured using the Luminex 100 system (Luminex, Austin, TX). The cytokine concentrations of cell culture supernatants were normalized against relative cell counts obtained by flow cytometry.

### Intracellular cytokine staining

The flow cytometric analysis of intracellular cytokine staining of transfected Th1 and Th2 polarized cells was performed after 7 days of priming. Shortly, NT, c-FLIP short and c-FLIP long siRNA transfected cells were activated and cultured under Th1 or Th2 polarizing conditions for 7 days after which the cells were harvested and washed with PBS. Half of the cells were restimulated with 5 ng/ml phorbol 12-myristate 13-acetate (PMA; Calbiochem, San Diego, CA) and 0.5 pg/ml ionomycin (Sigma-Aldrich) in Yssel's medium and the other half was incubated in Yssel's medium and used as an unstimulated control. After 2 h of incubation, 10 µg/ml brefeldin A (Alexis Biochemicals) was added and incubation was continued for another 3 h. Cells were washed twice with 0.5% BSA/PBS (w/v), 0.01% NaN_3_, fixed with 4% paraformaldehyde/PBS and permeabilized with 0.5% saponin/PBS. For staining of intracellular cytokines α-human-IFNγ-FITC (Invitrogen) and α-human-IL-4-PE (BD Pharmingen) were used. α-mouse-IgG1-FITC (Invitrogen) and α-rat-IgG1-PE (BD Pharmingen) were used as isotype controls. Cells were analyzed with the FACSCalibur system and analyzed with CellQuest Pro (both from BD Biosciences).

## Results

### c-FLIP expression is induced by TCR activation and Th1and Th2 driving cytokines

**Figure 1:**
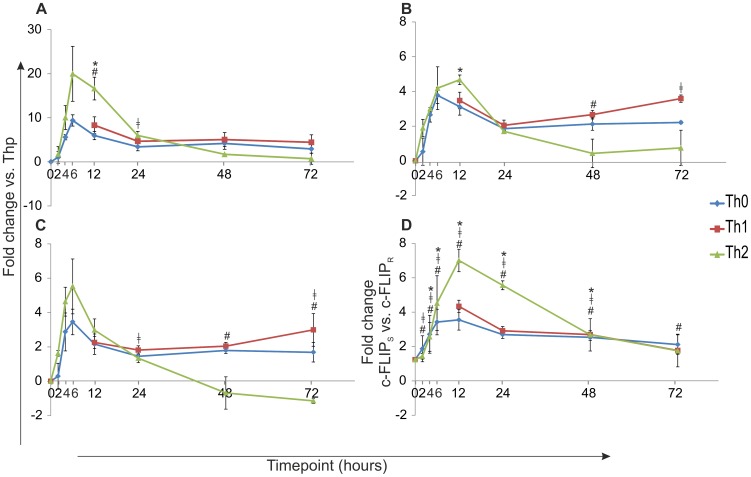
Induction of c-FLIP expression by TCR signaling and Th1/Th2 cytokines. Thp cells were isolated from cord blood and activated (Th0) or also stimulated with IL-12 (Th1) or IL-4 (Th2) and samples for real-time RT-PCR analysis were collected at indicated time-points. A–C. The expression of *c-FLIP_S_* (A), *c-FLIP_L_* (B) and *c-FLIP_R_* (C) mRNA during early Th cell differentiation. The graph represents the fold change (±SEM) for Th0, Th1 and Th2 calculated against Thp-sample. A–C. Statistical significance was calculated using paired student's t-test (* p<0.05; Th2 vs Th0, # p<0.05; Th2 vs Th1 and ‡ p<0.05 Th1 vs Th0). Results are calculated from three independent biological replicate cultures. D. The expression levels of *c-FLIP_S_* and *c-FLIP_R_* mRNA measured by real-time RT-PCR were compared with each other and are represented as fold change (paired student's t-test, p<0.05; *c-FLIP_S_* vs *c-FLIP_R_* all time-points in Th0 (#), all time-points except 72 h in Th1 cells (‡); and all time-points except 2 h and 72 h in Th2 cells (*)). Results are calculated from three independent biological replicate cultures. SEM =  standard error of mean.

To study the expression of three c-FLIP isoforms during early T helper cell differentiation in detail, human Thp cells were activated and polarized into Th0, Th1 and Th2 direction. The expression of c-FLIP isoforms was studied by real-time RT-PCR (Figure 1A–C and Figure S1). All isoforms were found to be rapidly up-regulated by TCR activation alone and the levels of *c-FLIP_L_, c-FLIP_S_* and *c-FLIP_R_* mRNA were increased already 2 h after the initiation of the culture compared with Thp cells. The TCR activation alone induced more efficiently the expression of *c-FLIP_S_* than either *c-FLIP_L_* or *c-FLIP_R_* expression. The Th2 polarizing condition further enhanced the TCR-induced up-regulation of c-FLIP isoforms and particularly the expression of *c-FLIP_S_* was more elevated in the Th2 cells at the early time-points of 2–24 h post cell activation (Figure 1A–C). The expression of all three isoforms peaked at 6 to 12 h after priming and decreased thereafter. To compare the levels of c-FLIP_S_ and c-FLIP_R_ isoforms, the relative mRNA levels of these isoforms were compared with each other (Figure 1D). The expression of c-FLIP_S_ was found to be up to 7 times higher than the expression of c-FLIP_R_ and this result was also statistically significant (p<0.05; all time-points in Th0, all time-points except 72 h in Th1 cells; and all time-points except 2 h and 72 h in Th2 cells).

### STAT6 is important for stable c-FLIP_S_ expression in Th2 cells

**Figure 2:**
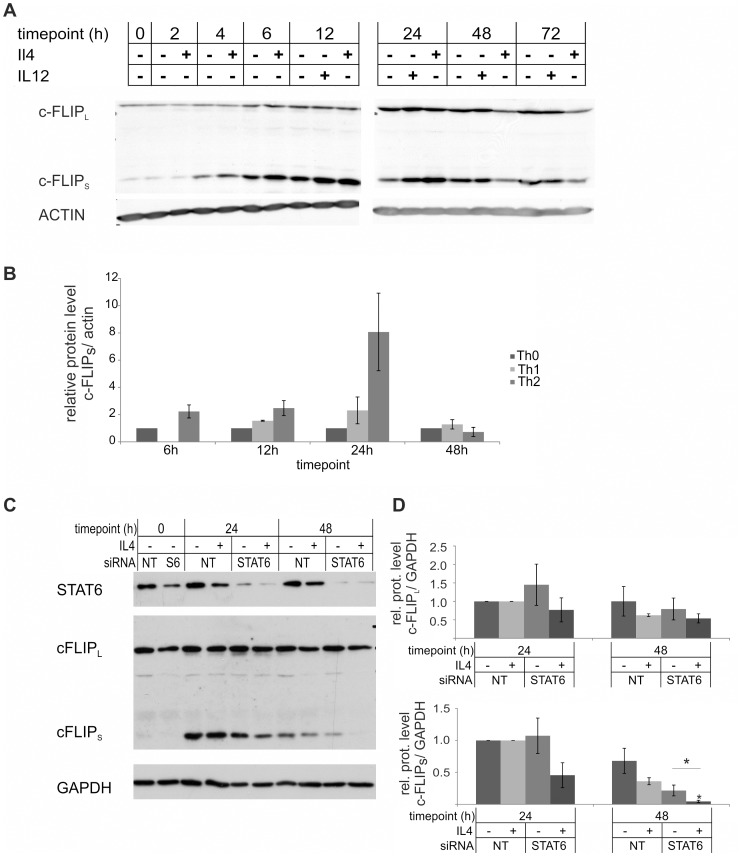
STAT6 is important for stable c-FLIP_S_ expression in Th2 cells. A. Thp cells were isolated from cord blood and activated (Th0) or also stimulated with IL-12 (Th1) or IL-4 (Th2) and samples for western blotting were collected at the indicated time-points. The panels show representative data from three independent biological replicate cultures. B. Bars represent the mean values (±SEM) of the relative levels of c-FLIP_S_ protein, obtained by quantifying and normalizing against the levels of β-ACTIN. The values of the Th0 samples were set as 1. Results were calculated from three independent biological replicate cultures. C. Freshly isolated Thp cells were transfected with STAT6 (S6) or non-targeting (NT) siRNA and polarized in Th0 or Th2 direction 20–24 h after transfection. Samples for western blotting were harvested at the indicated time-points. The panels show representative data of three biological replicate cultures. D. Bars represent the mean values (±SEM) of the relative levels of c-FLIP_L_ (upper panel) and c-FLIP_S_ (lower panel), obtained by quantifying and normalizing against the levels of GAPDH. The values of Th0 (24 h) and Th2 (24 h) were set as 1 and other Th0 and Th2 samples were compared to them. Results were calculated from three biological replicate cultures. Statistical significance was calculated using the paired student's t-test, * p>0.05. SEM =  standard error of mean.

To further characterize the expression of c-FLIP isoforms in human Th cells, we studied the kinetics of c-FLIP_S_ and c-FLIP_L_ on protein level during the early differentiation. c-FLIP_L_ is expressed in Thp cells whereas c-FLIP_S_ expression becomes visible soon after activation (Figure 2A). However, we could not detect c-FLIP_R_ isoform on protein level, which may be explained by its low expression level. This is also in line with the mRNA expression result that showed a much higher level of expression of c-FLIP_S_ than c-FLIP_R_ (Figure 1D). The expression of c-FLIP_S_ protein was more enhanced in Th2 cells than in Th1 cells (Figure 2B). Furthermore, our data indicated no clear difference on the expression of c-FLIP_L_ in either Th1 or Th2 polarizing conditions compared with Th0 cells (Figure 2A).

STAT6 is the major transcription factor driving Th2 differentiation and IL-4 signaling, and to study the role of STAT6 in the observed c-FLIP_S_ up-regulation in Th2 cells, we used an siRNA approach. We studied the kinetics of c-FLIP_S_ and c-FLIP_L_ expression in Thp cells transfected with STAT6 siRNA and activated in the presence or absence of IL-4. The protein level of c-FLIP_S_ was lower in STAT6 siRNA transfected Th2 cells compared with non-targeting (NT) siRNA transfected cells (Figure 2C) and thus the stable expression of cFLIP_S_ in the presence of IL-4 may, at least partly, be mediated by STAT6.

### Down-regulation of c-FLIP_S_ and c-FLIP_L_ affects the early polarization of human Th cells

**Figure 3:**
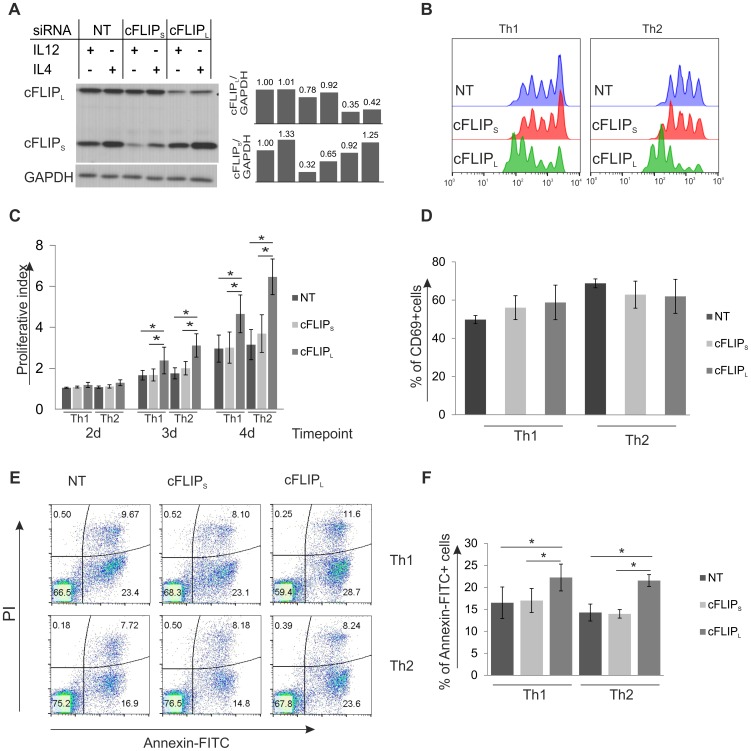
Knockdown of c-FLIP_L_ affects apoptosis and proliferation of Th cells. Freshly isolated Thp cells were transfected with c-FLIP_S_, c-FLIP_L_ or non-targeting (NT) siRNA, left to rest for 20–24 h and then activated and stimulated with IL-12 (Th1) or IL-4 (Th2). A. The knockdown efficiency of c-FLIP_S_ and c-FLIP_L_ siRNAs. Samples for western blotting analysis were harvested 24 h after priming. GAPDH was used as a loading control. Bars show relative levels of c-FLIP_L_ (upper panel) and c-FLIP_S_ (lower panel) obtained by quantifying and normalizing against the levels of GAPDH. The value of NT Th1 was set as 1. B. the knockdown of c-FLIP_L_ affects the proliferation of Th1 and Th2 cells. Transfected cells were left to rest for 20–24 h and then stained with CFSE and activated and stimulated with IL-12 (Th1) or IL-4 (Th2). The proliferation of CFSE stained cells was analyzed by flow cytometry at days 2, 3 and 4 after initiation of the culture. Histogram shows representative data of three independent biological replicate cultures at day 4. C. Bars show proliferative indexes calculated from three independent biological replicate cultures. Statistical significance was calculated using paired student's t-test, * p<0.05. D. Analysis of CD69 expression by flow cytometry at 24 h time-point. Results are calculated from three independent biological replicate cultures. E. c-FLIP_L_ knockdown Th cells have elevated levels of apoptotic cells. Transfected cells were left to rest for 24 h, cultured and activated in Th1 or Th2 conditions (as described above) followed by staining with Annexin-FITC and prodium iodide (PI). Representative data of three independent biological replicate cultures is shown. F. Bars represent the average of percentage of early apoptotic (Annexin-FITC+PI-) cells (±SEM). Results were calculated from three independent biological replicate cultures. Statistical significance was calculated using the paired student's t-test, * p<0.05. B–F. NT, cFLIP_S_ and cFLIP_L_ refer to the used siRNAs. SEM =  standard error of mean.

Since c-FLIP_S_ was found to be differentially expressed by IL-4 treatment during the early Th differentiation and c-FLIP_L_ was up-regulated by TCR activation, we further elucidated their possible roles in this process by using isoform specific siRNAs. Thp cells transfected with the c-FLIP_S_ or c-FLIP_L_ isoform specific siRNAs or NT siRNA were cultured in Th1 or Th2 polarizing conditions. Both of the c-FLIP isoform specific siRNAs were effectively knocking down their targets without affecting the expression of the other isoform (Figure 3A). Because of their role as regulators of apoptosis and T cell proliferation [25]–[27], [43], [44], we studied how the c-FLIP isoform specific knockdown affected the proliferation by using CFSE staining, activation by measuring CD69 expression and apoptosis by analyzing the number of annexin and propidium iodide (PI) positive cells. Interestingly, the c-FLIP_L_ knockdown cells were found to proliferate faster than the NT or c-FLIP_S_ siRNA treated cells (Figure 3B and 3C). The CD69 expression of transfected cells was analyzed by flow cytometry at 24 h time-point after cell activation (Figure 3D). The expression of CD69 was found to be similar between the c-FLIP siRNA and NT siRNA treated cells. Furthermore, cells treated with c-FLIP_L_ siRNA were more susceptible to apoptosis than control cells, but the number of dead cells was only slightly increased 24 h after activation (Figure 3E and 3F). Similar results were also obtained at 48 h time-point (data not shown). However, since the c-FLIP_L_ knockdown cells were also proliferating faster than the control cells, the total number of living cells was similar to that observed in NT siRNA treated cells.

**Figure 4:**
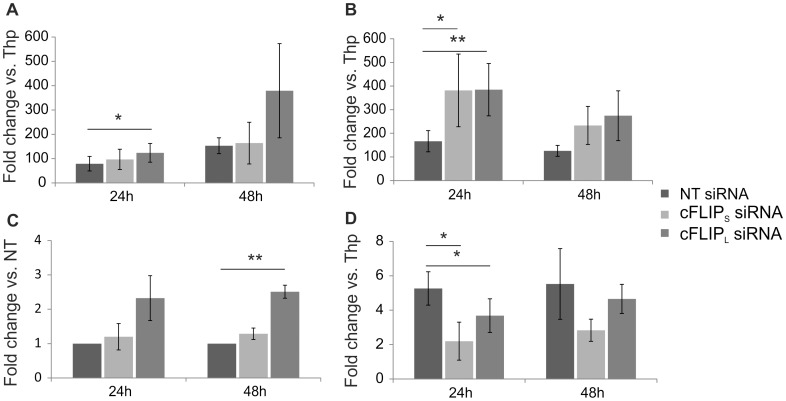
Knockdown of c-FLIP affects Th1 and Th2 markers. Freshly isolated Thp cells were transfected and cultured as described in Figure 3. Samples for real-time RT-PCR analysis were collected at indicated time-points. A. *IL12Rβ2* mRNA levels of transfected Th1 cells were analyzed. The graph shows average fold differences (±SEM) in the siRNA treated Th1 cells compared with Thp sample. The data is calculated from 3 independent cultures. B. *TBET* mRNA levels of transfected Th1 cells were analyzed. The graph shows average fold differences (±SEM) in the siRNA treated Th1 cells compared with Thp sample. The data is calculated from 5 independent cultures. C. *IFNG* mRNA levels of transfected Th1 cells were analyzed. The graph shows average fold differences (±SEM) in the c-FLIP_S_ and c-FLIP_L_ siRNA treated Th1 cells compared with non-targeting (NT) siRNA treated Th1 cells. The data is calculated from 4 independent cultures. D. *GATA3* mRNA levels of transfected Th2 cells were analyzed. The graph shows average fold differences (±SEM) in the siRNA treated Th2 cells compared with Thp sample. The data is calculated from 5 independent cultures. Statistical significances were calculated using the paired student's t-test, * p<0.05; ** p<0.01. SEM =  standard error of mean.

To investigate how the down-regulation of c-FLIP_S_ and c-FLIP_L_ influence Th1 and Th2 cell polarization, we first measured the expression of lineage specific markers *TBET, IL12RB2, IFNG* and *GATA3* at the mRNA level by real-time RT-PCR (Figure 4A–D). The mRNA expression of *TBET* was increased in response to the down-regulation of c-FLIP_S_ and c-FLIP_L_ compared with the control, whereas *IL12RB2* and *IFNG* were expressed at a higher level in c-FLIP_L_ knockdown Th1 cells compared with control cells. In Th2 cells, the expression of GATA3 mRNA was lower in cells transfected with c-FLIP_S_ or c-FLIP_L_ siRNAs than in the control cells. In summary, the down-regulation of c-FLIP_L_ or c-FLIP_S_ led to the up-regulation of Th1 marker genes (*TBET, IL12Rβ2* and *IFNG*) or *TBET* mRNA level, respectively, whereas a Th2 marker gene, *GATA3*, was down-regulated in response to the depletion of both c-FLIP isoforms.

### Down-regulation of c-FLIP_L_ results in increased IFNγ production by human Th1 cells

**Figure 5:**
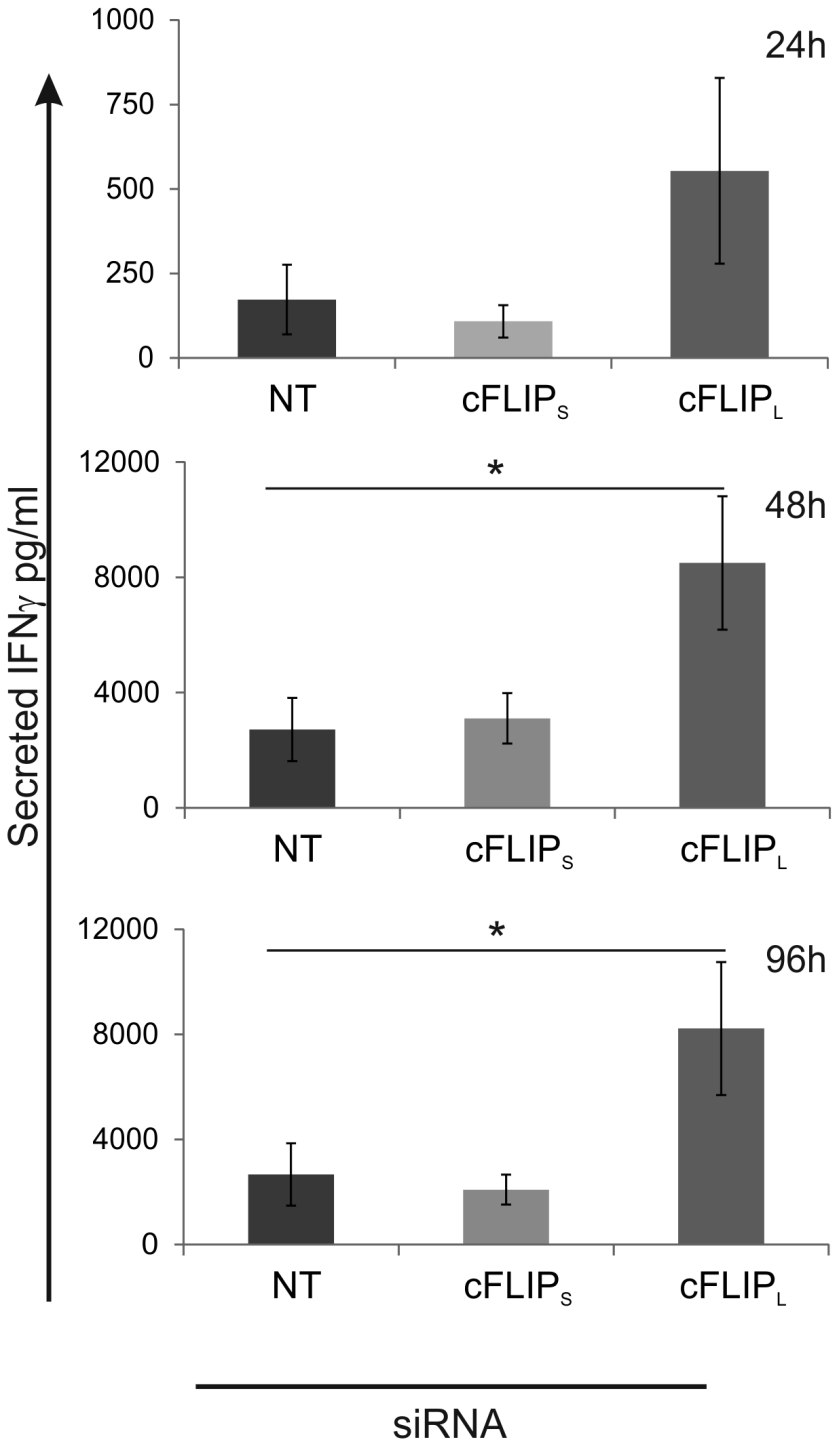
The down-regulation of c-FLIP_L_ results in the increase of secreted IFNγ by human Th1 cells. Freshly isolated Thp cells were transfected and cultured as described in Figure 3. Cell culture supernatants from Th1 polarized cells were collected at the indicated time-points, and the amount of secreted IFNγ produced by the cells was measured by cytokine assay. The bars represent the average secreted IFNγ in pg/ml (±SEM). Data is average of 5–6 independent cultures depending on time-point. Statistical significances were calculated using the paired student's t-test, *p<0.05. NT =  non-targeting, cFLIP_S_ and cFLIP_L_ refer to the used siRNAs. SEM =  standard error of mean.

**Figure 6:**
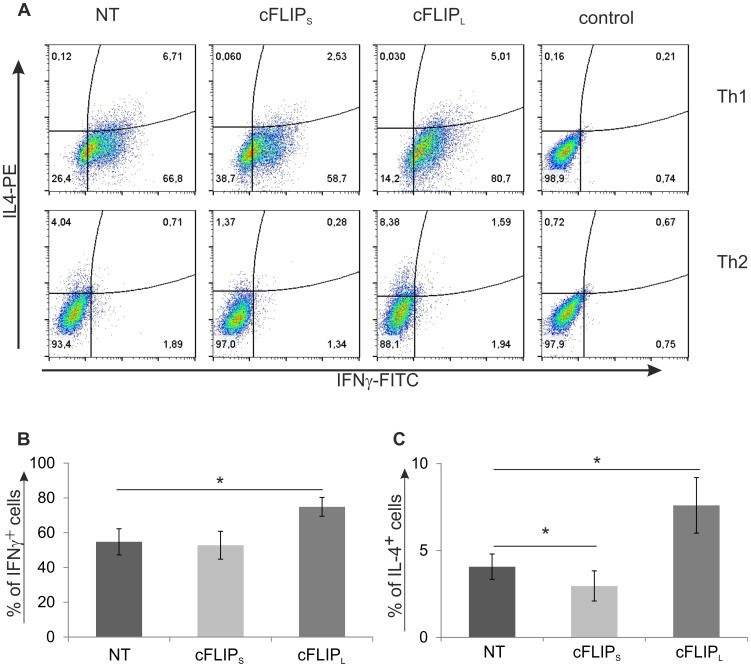
Knockdown of c-FLIP influences the cytokine production of Th1 and Th2 cells. Cells were cultured as explained in Figure 3. A. c-FLIP_S_, c-FLIP_L_ or non-targeting (NT) siRNA transfected cells were cultured in Th1 and Th2 polarizing conditions for 7 days. The cells were then harvested and restimulated or left unstimulated (control) and the levels of intracellular IFNγ and IL-4 were analyzed by flow cytometry. Dot plots show representative data of at least 7 independent biological replicate cultures. B. Bars represent the average percentage of IFNγ+ cells (±SEM) calculated from 7 independent cultures. C. Bars represent the average percentage of IL-4+ cells (±SEM) calculated from 8 independent cultures. B and C. Statistical significances were calculated using the paired student's t-test, *p<0.05. NT, cFLIP_S_ and cFLIP_L_ refer to the used siRNAs. SEM =  standard error of mean.

Cytokine production is one of the characteristic of the different Th cell subtypes. The hallmark cytokines produced by Th1 and Th2 cells are IFNγ and IL-4, respectively. Since c-FLIP knockdown altered the mRNA expression of Th1/Th2 marker genes during early polarization, we further characterized the effect of c-FLIP knockdown on IFNγ secretion by Th1 polarized cells using cytokine assay. To achieve this, Th cells transfected with c-FLIP_S_, c-FLIP_L_ or NT siRNA were cultured in Th1 polarizing conditions and the secreted IFNγ was measured from the cell culture supernatants at 1, 2 and 4 days after the initiation of culture (Figure 5). The amount of secreted IFNγ was more than 2-fold higher in c-FLIP_L_ knockdown Th1 cells compared with control. The effect of c-FLIP_L_ siRNA on IFNγ secretion was similar in all time points studied and statistically significant at days 2 and 4. Furthermore, we utilized intracellular cytokine staining to measure the IFNγ and IL-4 expression of Th1 and Th2 polarized cells. For this purpose the c-FLIP siRNA transfected cells were cultured in Th1 or Th2 polarizing conditions for 7 days, restimulated and the intracellular levels of IFNγ and IL-4 were measured by flow cytometry (Figure 6A). In line with the mRNA and cytokine assay results (Figure 1 and 5), the percentage of IFNγ producing cells was higher in c-FLIP_L_ knockdown Th1 cells compared with control cells (Figure 6B). Furthermore, our data showed that the number of IL-4 producing cells was higher in c-FLIP_L_ knockdown Th2 cells compared with control, whereas the knockdown of c-FLIP_S_ reduced the number of IL-4 producing cells (Figure 6C). The siRNA mediated c-FLIP knockdown was transient and could not be observed after 48 to 72 h of culture. Nevertheless, c-FLIP knockdown had a sustained effect on the cytokine expression in Th1 and Th2 cells, thus indicating that the early differentiation is altered by the knockdown of c-FLIP_S_ and c-FLIP_L_.

## Discussion

Previous studies have shown that in addition to apoptosis, CASPASE-8 and its regulator, c-FLIP, have a role in T cell activation, proliferation and differentiation [45]–[48]. In addition, the inhibition of Caspase-8 leads to an enhanced Th2 response in mice [29]. Although studied in mice, the role of c-FLIP in human Th cell differentiation has not been previously studied [31], [49]. In this study, we have characterized in detail the kinetics of c-FLIP expression during the early stages of human Th1 and Th2 cell differentiation and revealed that although all three c-FLIP isoforms are up-regulated by TCR activation, only c-FLIP_S_ isoform was selectively induced by the Th2 polarizing cytokine, IL-4. Most probably due to its low expression level we could not detect the expression of c-FLIP_R_ at protein level in this study. By using isoform specific siRNAs, we demonstrate that the knockdown of c-FLIP_L_ leads to the induction of Th1 marker genes, and to increased IL-4 production, whereas the knockdown of c-FLIP_S_ leads to the down-regulation of Th2 related genes *IL-4* and *GATA3*. Analysis of the impact of c-FLIP knockdown on cell viability and proliferation showed c-FLIP_L_ depleted cells to have elevated apoptosis and proliferation rates, whereas depletion of c-FLIP_S_ did not alter the cell viability or proliferation. Therefore, it seems that c-FLIP isoforms are both differentially expressed and have distinct roles during the early differentiation of human Th cells.

It has been shown that in human T cells the levels of c-FLIP_S_ are usually higher than the levels of c-FLIP_R_ [24], [50]. This is in line with our results showing up to 7-times higher expression of c-FLIP_S_ than c-FLIP_R_ at the mRNA level. In addition, STAT6, an important mediator in IL-4 signaling pathway, seems to be important for stable c-FLIP_S_ expression in Th2 cells. STAT transcription factors have been shown to bind to same binding sites and the usage of STAT may be cell type specific [51]. In fact STAT3, another STAT family member, has been shown to regulate c-FLIP_L_ in hepatocytes [52] so it is possible that STAT6 has a similar role in IL-4 induced Th2 cells to maintain a stable c-FLIP_S_ expression. Another possible candidate which may be involved in the expression of c-FLIP_S_ in Th2 cells could be NFAT2, which is a positive regulator of Th2 differentiation [53] and has been shown to selectively up-regulate the expression of c-FLIP_S_ [35]. c-FLIP proteins are well characterized for their role as regulators of apoptotic cell death. Transgenic mice overexpressing c-FLIP_L_ show resistance to both spontaneous and induced apoptosis [38], [43], [44]. c-FLIP_S_ can also act as an anti-apoptotic molecule by inhibiting Caspase-8 activation [25]. Our results are in line with the previous studies as we detected increased numbers of apoptotic cells after knockdown of c-FLIP_L_. As we did not detect any change in the viability or number of apoptotic cells in c-FLIP_S_ knockdown cells, it is possible that normal c-FLIP_L_ level present in the cell alone or together with low level of c-FLIP_S_ is enough to protect the cells from apoptosis. Thus it seems that the depletion of c-FLIP_L_ had bigger impact on the sensitivity of human Th cells to apoptosis than the depletion of c-FLIP_S_ in these cells. c-FLIP_L_ transgenic mice show decreased level of proliferation, although with suboptimal levels of anti-CD3 activation, c-FLIP_L_ transgenic T cells proliferate faster than wild-type T cells [38], [43], [44]. In addition, T cell proliferation is suppressed in human primary T cells treated with Caspase-8 inhibitors [54], [55] and both human and murine T cells deficient for functional Caspase-8 [46], [47]. Thus, our observation that knockdown of c-FLIP_L_ led to increased proliferation of both Th1 and Th2 cells is in line with the previous studies. On the other hand, the c-FLIP_S_ transgenic mice do not show difference in cell proliferation compared with control [56] similar to the findings on c-FLIP_S_ knockdown T cells in our study. On the basis of our results it seems that c-FLIP_L_ influenced both the apoptosis and proliferation of human Th cells whereas c-FLIP_S_ did not have an effect.

In line with our results showing that the knockdown of c-FLIP_L_ induces IFNγ production and up-regulates *TBET* expression, the opposite, i.e. decreased levels of IFNγ and TBET expression, were detected in transgenic mice expressing c-FLIP_L_ in the T cell compartment [31]. However, contradictory to our data showing higher IL-4 production in c-FLIP_L_ depleted Th2 cells, c-FLIP_L_ transgenic mice have also elevated levels of GATA3 and Th2 cytokines [31], [49]. In our study the c-FLIP_L_ knockdown cells showed faster proliferation rate than control or c-FLIP_S_ knockdown cells. It has been shown that increased proliferation of T cells correlates with elevated levels of produced cytokines [57], [58]. Thus the enhanced proliferation may explain the increased numbers of both IFNγ and IL-4 producing cells observed after knockdown of c-FLIP_L_. Nonetheless, our observation indicating that the down-regulation of c-FLIP_L_ in human Th cells promotes Th1 differentiation is in line with the mouse studies. Wu *et al*. [31] demonstrated in c-FLIP_L_ transgenic mice that the decreased levels of IFNγ and increased Th2 cytokines were at least partly independent from each other suggesting that in our data it is also possible that different mechanisms are driving the elevated Th1 response and the increased IL-4 production [31], [49]. In addition, CASPASE-8 inhibition in mouse Th cells leads to the elevated expression of GATA3 and IL-4 [29], which is in line with the decreased IL-4 and *GATA3* expression observed in c-FLIP_S_ knockdown Th2 cells in this study. Furthermore, the decreased levels of *GATA3* expression and IL-4 production cannot be explained by augmented apoptosis since the c-FLIP_S_ knockdown cells did not show elevated level of apoptosis.

Two c-FLIP isoforms have been shown to activate both ERK signaling and NF-κB signaling in response to activation in Jurkat T cells overexpressing c-FLIP_S_ or c-FLIP_L_, respectively [22]. Thus possible mechanisms by which the c-FLIP proteins might alter the gene expression of differentiating Th cells could be the ERK pathway and NF-κB pathway [59]–[61]. Other signaling pathways including p38 MAPK and AP-1 transcription factors have also been linked to c-FLIP activity and expression [31], [62], [63]. It is thus possible that modulation of ERK, NF-κB or some other signaling pathway by c-FLIP_S_ and c-FLIP_L_ may result, at least partly, in the changes observed in Th1 and Th2 cell differentiation in response to knockdown of c-FLIP_L_ and c-FLIP_S_ in this study.

In summary, we have demonstrated that c-FLIP isoforms, c-FLIP_S_ and c-FLIP_L_, are differentially expressed during the early polarization of human Th1 and Th2 cells. In addition, by using an siRNA approach we were able to show that the knockdown of c-FLIP_L_ and c-FLIP_S_ had distinct effects on Th1/Th2 cell differentiation. c-FLIP_L_ knockdown led to enhanced Th cell proliferation and cytokine production by both Th1 and Th2 cells, while the knockdown of c-FLIP_S_ reduced the expression of genes important for Th2 polarization. This study provides new insight into the roles of c-FLIP proteins in Th cell differentiation.

## Supporting Information

Figure S1 **The polarization of the cultures used in** **Figures 1** **and** **2A–B** **.** The polarization of the cultures was confirmed by RT-PCR. Graphs show average fold change vs. Thp sample calculated from three independent cultures. Error bars represent standard error of mean (SEM).
(PDF)
