# Supplementary material for: The Regulation and Role of c-FLIP in Human Th Cell Differentiation
Source: PLoS One. 2014 Jul 14;9(7):e102022. doi: 10.1371/journal.pone.0102022 (PMC4096760; doi:10.1371/journal.pone.0102022)
Supplement: Figure S1 — The polarization of the cultures used in Figures 1 and 2A–B . The polarization of the cultures was confirmed by RT-PCR. Graphs show average fold change vs. Thp sample calculated from three independent cultures. Error bars represent standard error of mean (SEM). (PDF) [file pone.0102022.s001.pdf]

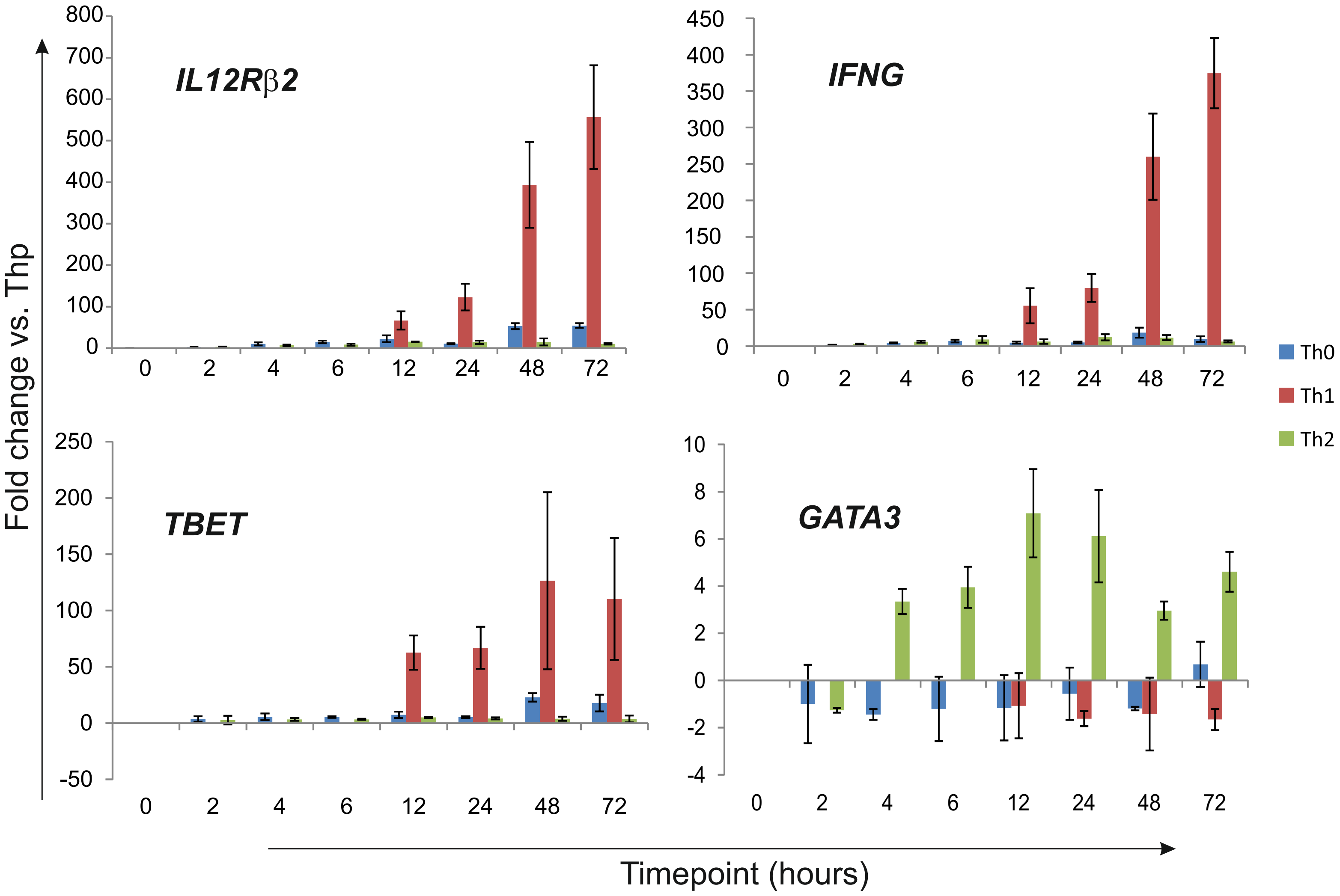

Figure S1. The polarization of the cultures used in Figures 1 and 2A-B. The polarization of the cultures was confirmed by RT-PCR. Graphs show average fold change vs. Thp sample calculated from three independent cultures. Error bars represent standard error of mean (SEM).
